# Masculinity in Caregiving: Impact on Quality of Life and Self-Stigma in Caregivers of People with Multiple Sclerosis

**DOI:** 10.3390/healthcare13030272

**Published:** 2025-01-30

**Authors:** Arturo Alvarez-Roldan, Félix Bravo-González

**Affiliations:** 1 Department of Social Anthropology, University of Granada, 18071 Granada, Spain; 2 Federation of Multiple Sclerosis Associations of Andalusia (FEDEMA), Avda. de Altamira, 29-B1-11-Acc. A., 41020 Sevilla, Spain; fbravo5@yahoo.es

**Keywords:** multiple sclerosis, male caregivers, gender, quality of life, burden, self-stigma

## Abstract

Background/Objectives: This study aims to examine the impact of the caregiving role on quality of life, perceived burden, gender dissonance, and self-stigma among male caregivers of individuals with multiple sclerosis (MS) in Andalusia, Spain. Methods: A total of 44 male caregivers participated, completing questionnaires on sociodemographic and functional characteristics of the persons with MS (PwMS) they cared for. Measures included the Caregiving Tasks in Multiple Sclerosis Scale (CTiMSS), the Zarit Burden Interview (ZBI), quality of life (WHOQOL-BREF), gender perception, and self-stigma. Data analysis employed ANOVA and regression analyses to assess the relationship between perceived burden and quality of life. Results: Male caregivers experienced significant burden, particularly in instrumental and social–practical caregiving tasks, with a mean score of 23.9 on the ZBI. Perceived burden was inversely related to quality of life, notably impacting physical and psychological health. Sixteen percent of caregivers reported cognitive self-stigma, though without affective or behavioral manifestations. Gender dissonance was observed, as men often perceived certain caregiving tasks as feminine; however, many integrated this role within their masculine identity. Conclusions: Male caregivers of PwMS face the dual burden of caregiving demands and traditional gender expectations. While gender dissonance and low levels of self-stigma were observed, most caregivers adapted by integrating caregiving into their identity. These findings highlight the need for tailored interventions to address their unique challenges and enhance their well-being.

## 1. Introduction

Multiple sclerosis (MS) is a chronic, immune-mediated, demyelinating disease affecting the central nervous system, causing a wide range of physical, cognitive, and emotional symptoms. Although the disease course can vary significantly, the most common form is relapsing–remitting MS, characterized by episodes of relapse followed by remissions, which in many cases leads to chronic progression [1]. People with multiple sclerosis (PwMS) often face severe limitations in their daily lives, ranging from motor and sensory difficulties to psychological, sexual, and bladder issues. These limitations not only affect patients but also those around them, especially family caregivers (FCs), who take on a large portion of daily caregiving responsibilities [2].

The impact of multiple sclerosis on FCs is profound, as they must provide assistance in various areas, including mobility, personal care, domestic tasks, and emotional support [3]. The progressive dependency of PwMS places a significant burden on FCs, who find that the time and energy they dedicate to caregiving tasks interferes with their physical and mental health, as well as their quality of life. Over time, FCs may experience high levels of stress, burnout, and, in many cases, symptoms of depression, impacting their overall well-being and their ability to cope with the situation [4,5,6].

While much of the research on informal caregivers has focused on women due to their traditionally assigned role in caring for sick individuals, studies on MS reveal an interesting peculiarity, namely that a significant percentage of FCs are men. In Spain, it is estimated that between 37% and 49% of informal caregivers of PwMS are men [7,8,9], a higher proportion than observed in other chronic illnesses. This phenomenon provides a valuable opportunity to explore how the experience of caring for a family member with a chronic illness may differ for men, as traditional gender roles do not typically associate informal care with masculinity [10].

The fact that nearly half of informal caregivers for PwMS are men raises several key questions. Unlike other chronic conditions where caregiving is more feminized, men who take on this responsibility face not only the physical and emotional demands of caregiving but also the challenges of a society that has historically assigned these tasks to women. This creates a potential conflict between social expectations of masculinity and the demands of the caregiving role, which can lead to a form of “gender dissonance” [11]. That is, the act of caregiving may conflict with traditional gender norms that view these activities as feminine, potentially increasing the risk of self-stigma in these men.

In this context, self-stigma refers to the internalization of negative stereotypes about the caregiving role, which could impact not only the mental health of male caregivers but also their identity and perception of their own masculinity. The sense that their actions are incongruent with social expectations of masculinity may lead them to perceive their role as a source of shame or inadequacy, affecting their emotional well-being and quality of life. Meanwhile, caregiving demands continue to place a significant burden on them, exacerbating negative effects on their health and social relationships.

This research focuses on analyzing the experience of male caregivers of PwMS, a group that, despite its growing importance, has received little attention in the scientific literature. We hypothesize that these men take on the caregiving role voluntarily, facing the same practical difficulties as women in terms of time management, physical effort, and emotional support but with the added challenge of feeling a conflict between caregiving and their masculine identity due to the cultural perception that associates caregiving with femininity.

The objectives of this study are as follows:

1. To identify the main caregiving tasks performed by male caregivers and explore their motivations.
2. To examine the perceived burden of caregiving tasks, its impact on their quality of life, and the relationship between the functional characteristics of PwMS and the caregivers’ burden and quality of life.
3. To analyze whether caregiving leads to gender dissonance or self-stigma, providing insights for designing interventions that enhance their well-being and support their essential role.

## 2. Methods

### 2.1. Participants and Procedure

This was an observational descriptive study in which we analyzed a series of cases. We recruited a purposive sample of 44 male caregivers residing in Andalusia, Spain. The inclusion criteria were as follows: being male, of legal age, a family caregiver for a person with MS, and providing informed consent. The exclusion criteria were not being the primary caregiver or receiving any form of payment for providing care. One of the authors, who has been a caregiver for his wife with MS for over 20 years and is a member of the Board of Directors of the Federation of Multiple Sclerosis Associations of Andalusia, utilized his connections within the associative network to contact associations in the eight provinces of the Andalusian region and invite male caregivers to participate in the study. Participation was voluntary and anonymous. Participants were informed about the research objectives and provided informed consent to complete the questionnaire (average completion time: 30 min). Data collection was conducted through personal interviews (either in person or by phone) without offering financial incentives to participants.

### 2.2. Instruments

1. Sociodemographic questionnaire: This questionnaire collected sociodemographic information about the caregivers and PwMS, including clinical and functional data for the latter. Additionally, it included two open-ended questions about caregiving motivations and the perceived quality of care compared to that provided by a woman. The first question was “How, why, and what were your motivations to start caring for a family member with multiple sclerosis?” The second was “Do you consider the quality of the care you provide to be inferior to that which would be provided by a woman?”

2. Caregiving Tasks in Multiple Sclerosis Scale (CTiMSS): This scale was used to assess the caregiving tasks performed by caregivers. The Spanish-translated scale includes 24 items, divided into four domains, namely activities of daily living (ADLs), psycho-emotional support, instrumental help, and social–practical support. Caregivers rated the level of help on a 5-point Likert scale, ranging from “Never” (0) to “Always” (4) [12]. The internal consistency of the scale in this study was excellent, with a Cronbach’s alpha of 0.93.

3. Zarit Caregiver Burden Interview (ZBI): The Spanish version was used to measure perceived burden among caregivers [13]. This 22-item questionnaire, with responses on a 0–4 Likert scale, evaluates areas such as physical health, psychological well-being, financial situation, and social life of the caregiver [14]. The Spanish version has demonstrated excellent internal consistency, with a Cronbach’s alpha between 0.83 and 0.94 [13,15].

4. WHOQOL-BREF Quality of Life Questionnaire: Developed by the WHO and adapted into Spanish [16,17], this questionnaire evaluates quality of life through 26 questions across four domains, including physical health, psychological health, social relationships, and environment. Higher scores indicate better quality of life. The Spanish version has satisfactory psychometric properties, including acceptability, internal consistency, and evidence of convergent and discriminant validity [18,19].

5. Gender perception in caregiving tasks: Caregivers rated whether they considered caregiving tasks to be more “masculine” or “feminine” on a 5-point Likert scale, from “Entirely masculine” (1) to “Entirely feminine” (5). This type of scale is easy to understand and has been used successfully in other studies to determine gender identity [20]. In this study, the internal consistency of the scale was good, with a Cronbach’s alpha of 0.82.

6. Self-Stigma Scale—Short Form (SSS-SF): To measure caregivers’ internalized stigma, this 9-item questionnaire used a 4-point Likert scale without a neutral option. It evaluates three dimensions of self-stigma, namely cognitive, affective, and behavioral dimensions. This scale has been used previously in studies with people who belong to minorities or have mental illnesses [21]. In this study, the internal consistency of the scale was good, with a Cronbach’s alpha of 0.77. For our analysis, we dichotomized the Likert scale values as follows: 1 and 2 = Yes; 3 and 4 = No.

### 2.3. Data Analysis

Data analysis was performed using R software (version 4.2.3). Descriptive and univariate analyses were conducted on the sociodemographic and functional variables of the caregivers and PwMS, as well as on the different scales used (CTiMSS, Zarit, WHOQOL-BREF, gender perception, and self-stigma). Missing values were imputed using the mean value of the variable or regression results in the case of the gender perception scale.

An ANOVA was used to analyze the relationship between perceived burden and quality of life, with Bartlett’s test used to verify the homogeneity of variances. A *p*-value < 0.05 was considered statistically significant, and effect size was evaluated using eta squared (η²).

Additionally, regression analyses were performed to examine the relationship between two indicators of PwMS functionality (type of MS and disability level) and two dependent variables, which were perceived burden (measured with ZBI) and the general quality of life of the caregiver (measured with WHOQOL).

## 3. Results

### 3.1. Characteristics of Caregivers and PwMS

Table 1 presents the characteristics of the 44 male caregivers who participated in the study, all residents of Andalusia, Spain. The average age of caregivers was 58.4 years. In terms of educational level, primary and secondary studies predominated (45.4%), with a lower proportion holding university degrees (27.3%). Approximately half of the participants were working, while the rest were retired or unemployed. The majority of caregivers (86.4%) were married or in a domestic partnership, and 80.5% were responsible for caring for their spouse or partner. The average duration of caregiving was 12.9 years. Almost all (95.5%) believe that the care they provide is of the same quality as that provided by a woman.

Regarding PwMS, most were women (87%), with an average age of 51.6 years, primarily (80.5%) wives or partners of the male caregiver. They commonly had relapsing–remitting (56.8%) or secondary progressive MS (38.6%).

In Spain, disability assessment is carried out by the public administration following technical guidelines. A multidisciplinary team, including at least a doctor, psychologist, and social worker, evaluates the individual’s physical, mental, and sensory conditions. Social factors, such as family environment and employment, educational, and cultural situations, are also considered. Disability is graded in percentages and categorized into three levels of <33%, 33–65%, and >65%. Dependency recognition, which is required to access public assistance, is governed by Law 39/2006 on the Promotion of Personal Autonomy and Care for Dependent Persons. This process involves a complex administrative procedure, which can be challenging to navigate. It is important to note that having a recognized degree of disability does not automatically entitle an individual to receive dependency-related benefits in the same proportion.

In this study, nearly three out of four people with PwSMS had reduced mobility, and 95.5% had recognized disabilities. However, only 45.5% qualified as dependent and were entitled to assistance.

### 3.2. Caregiving Tasks Performed by Caregivers

Table 2 shows the frequency of participation of FCs in various caregiving tasks, evaluated on a 5-point Likert scale where 0 indicates “Never” and 4 indicates “Always”. Caregivers provided higher levels of support in tasks related to instrumental and social–practical caregiving. The tasks in which they provided the most help included grocery shopping (*M* = 3.0; SD = 1.2), transportation (*M* = 2.9; *SD* = 1.4), housework (*M* = 2.8; *SD* = 1.3), emotional support (*M* = 2.7; *SD* = 1.0), meal preparation (*M* = 2.6; *SD* = 1.4), companionship (*M* = 2.5; *SD* = 1.3), and physical exercise (*M* = 2.3; *SD* = 1.3). These tasks entail a high level of physical and organizational responsibility on the part of the caregivers, reflecting a strong commitment to daily management and the well-being of the dependent individual.

On the other hand, tasks where less support is provided are primarily concentrated in ADL care. Among the activities receiving the least help are feeding (*M* = 0.7; *SD* = 1.1), managing incontinence (*M* = 0.9; *SD* = 1.5), administering medications (*M* = 1.2; *SD* = 1.5), dealing with memory loss (*M* = 1.2; *SD* = 1.1), keeping the patient occupied (*M* = 1.3; *SD* = 1.2), and assisting with toilet use (*M* = 1.4; *SD* = 1.5). These results suggest that caregivers are less involved in activities that may relate to the patient’s desire for greater autonomy or the use of devices or external resources that facilitate care.

From these results, it appears that male caregivers provide significant help in tasks traditionally considered “feminine”, such as meal preparation, housework, shopping, and providing emotional support. This behavior challenges conventional gender stereotypes, showing that male caregivers take on active roles in tasks historically associated with women.

Conversely, the tasks in which they offer less help are primarily basic care tasks, such as feeding and managing incontinence. This pattern may reflect a lower willingness or need to engage in more intimate or routine activities that are traditionally perceived as part of female caregiving.

Overall, the fact that male caregivers provide substantial assistance with activities like cooking and shopping suggests greater flexibility in role-taking within caregiving. Male caregivers assume responsibilities in both instrumental and emotional areas, which is crucial for the well-being of dependent individuals, who are mostly women.

### 3.3. Motivations and Care Evaluation

Caregivers’ responses to the question of how and why they decided to start caring for a family member with multiple sclerosis underscore the voluntary nature of their decision and the importance of family ties and affinity. Two-thirds made the decision spontaneously and voluntarily, with most doing so from the outset. Eighteen percent indicated that they took on caregiving gradually as the symptoms of the disease worsened. Fourteen percent said they did so out of “responsibility”, while only two caregivers felt obligated and another two did so out of necessity.

Regarding motivations, the primary reason was that the ill person was a family member (52.3%): “she’s my wife”, “we live together as a couple”, “he’s my son, and I want the best quality of life for him”, “she’s my sister”, “taking care of my mother as she took care of me”, “I’m the head of the family”, “preserving the family project”, “the family looks after its members”, “protecting the family”. One in three caregivers cited affective and sentimental reasons as their main motivation for becoming a caregiver.

Most rated the quality of the care they provided as equal to or better than that provided by a woman. Statements included “No one’s going to take care of her better than I can; I know her very well”, “both genders can perform almost all caregiving tasks”, “I take on any task that’s needed and never turn my back on it, no matter how unpleasant it is”, “I believe I do it as well as any woman”, “care is not a matter of gender”, “the level of care and ability doesn’t depend on gender”, “I feel equally or even better prepared than a woman”, “what a woman can do, a man can also do”, “anything a woman could do, I do”, “I’m stronger as a man, and since I also have MS, I understand it well”, “if you have an interest, you learn, and quality comes with practice”. Only two caregivers noted their lack of experience, stating that a woman would do better: “women adapt better”, “I don’t know much about housework”.

### 3.4. Perceived Burden

The mean score on the Zarit Burden Interview for the entire sample was 23.9 (*SD* = 10.6). Table 3 shows the results broken down by burden level. One in three FCs (31.8%) reported mild-to-moderate burden as a result of their caregiving. Fifty-six percent reported a moderate-to-intense burden and would need to modify their caregiving approach to the dependent person. Finally, 11.4% of caregivers experienced a high level of burden, which carries an elevated risk of developing illnesses, especially depression and anxiety [6,8,13,22].

### 3.5. Perceived Quality of Life

In general, caregivers rated their quality of life and health as average, neither good nor bad, with mean scores of 54 and 52.8 out of 100, respectively (Table 4). However, one in three perceives their quality of life as very poor (25 points). When assessing the different components of health and quality of life, scores rose to 60 or above in areas such as physical health, psychological health, and environmental quality. In contrast, the rating of social relationships drops to 53 points, consistent with their general perception of quality of life and health.

The dimension rated lowest in their quality of life relates to social relationships, which include indicators such as personal relationships, social support, and sexual activity.

### 3.6. Relationship Between Perceived Burden and Quality of Life

To assess the relationship between perceived burden and quality of life, Zarit test results were categorized into four levels, as none-to-mild, mild-to-moderate, moderate-to-severe, and severe. Subsequently, ANOVAs were conducted to compare WHOQOL scores across these groups.

Results show significant differences between the burden level and several quality of life dimensions, as detailed below (Table 5). Caregivers with severe burden reported significantly lower quality of life than those with no or mild burden. The effect size (η²) was considerable, explaining between 18% and 35% of the variance in quality of life.

Specifically, overall quality of life declines drastically, from an average of 60.7 points among caregivers with mild-to-moderate burden to 56 points for those with moderate-to-severe burden and to 25 points for those reporting severe burden. Similarly, dimensions of physical health, psychological health, and environmental quality also show a significant decrease among caregivers with higher burden levels. However, no significant differences were found in the scores for social relationships or general health, suggesting that these areas may not be as affected by perceived burden.

These findings underscore the importance of addressing caregiver burden not only from an emotional perspective but also considering its impact on physical health, psychological well-being, and environmental perception. Caregivers with high burden levels need interventions to improve their overall well-being and prevent deterioration in their quality of life.

### 3.7. Relationship Between the Dependent Person’s Functionality and the Caregiver’s Burden and Quality of Life

Regression analyses were conducted to examine the relationship between indicators of the dependent person’s functionality (type of MS and disability level) with the perceived burden (ZBI) and the caregiver’s overall quality of life (WHOQOL1). Results showed that both regression models were statistically significant.

In the first model, where perceived burden (ZBI) was the dependent variable, the type of multiple sclerosis was found to be a significant predictor (see Table 6). Specifically, compared to PP MS, RR MS showed a significant negative association with perceived burden, *b* = −23.89, *p* < 0.01, 95% CI [−40.57; −7.31], with an *sr²* = 0.017, 95% CI [−0.03; 0.37]. SP MS was also a significant negative predictor, *b* = −19.37, *p* < 0.05, 95% CI [−36.83; −1.90], with an *sr²* = 0.10, 95% CI [−0.06; 0.26]. Disability level did not reach statistical significance. The model explained 22% of the total variance (*R²* = 0.220, 95% CI [0.00; 0.37]).

In the second model, with overall quality of life (WHOQOL1) as the criterion, both MS type and disability level were significant predictors (see Table 7). Using PP MS as a reference, RR MS was a significant positive predictor, *b* = 36.22, *p* < 0.05, 95% CI [6.87; 65.56], with an *sr²* = 0.10, 95% CI [−0.04; 0.23], as was SP MS, *b* = 37.78, *p* < 0.05, 95% CI [6.88; 68.69], with an *sr²* = 0.09, 95% CI [−0.04; 0.23]. On the other hand, having more than 65% disability was a significant negative predictor of quality of life, *b* = −39.84, *p* < 0.05, 95% CI [−70.33; −9.34], with an *sr²* = 0.11, 95% CI [−0.04; 0.25]. The model explained 40.4% of the total variance (R² = 0.404, 95% CI [0.12; 0.54]).

### 3.8. Gender Dissonance and Self-Stigma

To explore the relationship between the male caregiving role and the gender perception of caregiving tasks, two aspects were evaluated, the perceived femininity/masculinity of the tasks and the level of self-stigma among male caregivers.

#### 3.8.1. Task Femininity

Table 8 presents the average scores obtained for each task on a scale from one (entirely masculine) to five (entirely feminine). Scores higher than three indicate that tasks are perceived as more feminine than masculine.

The results show that 12 tasks are perceived as feminine (*M* > 3), seven as masculine (*M* < 3), and five as neutral (*M* = 3). Basic daily life care and instrumental care are predominantly perceived as feminine, while socio-practical care is mostly seen as masculine, with the exceptions of emotional support and keeping the patient occupied.

This gendered division of caregiving tasks aligns with cultural stereotypes prevalent in Andalusian society [23,24]. However, when considering the standard deviation, most tasks tend to be perceived as gender neutral. This suggests that caregivers in this study may have responded based on contemporary societal perceptions—ones that view traditional gender norms as outdated—rather than their personal beliefs, which are likely influenced by their age and the societal context in which they were raised.

It is noteworthy that caregivers provide greater support in instrumental and socio-practical care (see Table 2), suggesting that gender dissociation primarily occurs in the former. Five of the tasks requiring the most time investment are considered feminine, including shopping, performing household chores, preparing meals, providing emotional support, and keeping the dependent person occupied.

#### 3.8.2. Self-Stigma Among Male Caregivers

Table 9 summarizes the results of the shortened self-stigma scale by Mak and Cheung [21], which evaluates three components of self-stigma, namely cognitive, affective, and behavioral components. Each item was scored using a 4-point Likert scale (1: strongly disagree; 2: disagree; 3: agree; 4: strongly agree) with no neutral option. The score for each of the three components corresponds to the average of its three items. Finally, we dichotomized the Likert scale values as follows: 1 and 2 = Yes; 3 and 4 = No.

Overall, only 16% of caregivers acknowledged experiencing cognitive self-stigma. This suggests that while gender dissonance might generate cognitive discomfort, it does not significantly affect affective or behavioral levels. Male caregivers do not appear to perceive caregiving as socially stigmatizing.

## 4. Discussion

Caregiving is a complex and multidimensional role that often intersects with issues of gender and masculinity. This discussion explores the findings of our study in two parts; first, by analyzing the caregiving responsibilities, the burdens they impose, and their impact on the quality of life for male caregivers of PwMS; second, by examining how male caregivers perceive the gendered nature of their caregiving tasks, including the strategies they use to navigate potential conflicts with traditional norms of masculinity.

### 4.1. Caregiving Responsibilities, Burden, and Quality of Life

This study reveals that male caregivers of PwMS play significant roles in instrumental tasks, such as shopping, transportation, housework, and meal preparation, as well as providing emotional support—roles that challenge traditional gender stereotypes. In contrast, they provide less assistance with personal autonomy tasks (e.g., toileting, intimate hygiene, dressing, feeding, or medication administration), which are often retained by the person with MS. This division highlights the boundaries of autonomy that individuals with MS seek to preserve, while household tasks are among the first to be delegated [11].

The time caregivers dedicate to these roles varies by sex and gender, as consistently shown in the literature. Women are more likely to devote over 20 h per week to caregiving, often at the expense of their employment, while male caregiving increases in older age and retirement, particularly in wealthier countries where gender labor gaps are narrower [25,26,27]. In our study, most male caregivers were older adults, with 45.5% aged 60 or above, 43.2% retired, and 11.4% unemployed. This aligns with previous research conducted in Spain indicating that men engaged in informal caregiving tend to be older than women and mostly care for their wives or partners [28,29,30,31].

Caregiving frequently imposes a burden that negatively affects caregivers’ quality of life across physical, psychological, and social domains [32,33]. Male caregivers in this study experienced varying levels of burden, with 68.2% reporting moderate-to-severe burden. Although their average burden level (*M* = 23.9) was comparable to previous studies [32,34,35,36,37], the impact on their overall quality of life was evident, especially in the domain of social relationships, aligning with traditional masculine concerns about personal connections and sexual intimacy [38,39].

Sex and gender differences in caregiving burden remain a topic of ongoing research [40]. Women often report higher ZBI scores, more mood disorders, and greater challenges in financial and occupational domains, while men are more likely to face physical health issues [28,29,30,31,32,41]. However, some studies suggest that when other factors, such as caregiving intensity and available support, are accounted for, gender differences may diminish [42].

Several studies have also pointed to a significant correlation between the type of MS and the burden experienced by caregivers, with the highest burden associated with PP MS, followed by SP MS, and RR MS [31,34]. Our findings support this relationship. Moreover, both the type of MS and the level of disability influence impacted quality of life, as also noted in other studies [6,7,22].

### 4.2. Masculinity and the Gendered Nature of Caregiving

Male caregivers in this study frequently acknowledged the traditionally feminine nature of many caregiving tasks they perform, such as cooking, shopping, and personal care. While this acknowledgment could create a sense of gender dissonance [43,44], few caregivers reported significant self-stigma or conflict with masculine norms. Instead, they employed various strategies to reconcile caregiving with their sense of masculinity.

1. Integration into traditional masculine roles: Some caregivers reframed their caregiving responsibilities as an extension of their protective and provider roles within the family [39,43,44,45]. For them, caregiving reinforced their masculine identity by demonstrating strength, resilience, and duty. This approach allowed them to align caregiving with traditional norms of masculinity, presenting themselves as steadfast heads of their households.
2. Adopting a gender-neutral perspective: Other caregivers approached caregiving from a gender-neutral standpoint, asserting that caregiving tasks are not inherently tied to masculinity or femininity [46,47]. They rejected the idea that caregiving competence depends on gender, as reflected in statements like “What a woman can do, a man can do”.
3. Redefining masculinity through caregiving: A third group went further, challenging traditional gender norms by embracing caregiving as a core part of their masculine identity [39,46]. These caregivers asserted that the quality of care they provided was equal to or superior to that of women, emphasizing their commitment, deep understanding of the dependent individual’s needs, and willingness to take on any caregiving task, no matter how demanding.

These strategies illustrate different approaches male caregivers use to navigate the intersection of caregiving and masculinity, whether by aligning caregiving with traditional roles, neutralizing its gendered nature, or redefining masculinity to encompass caregiving [46,47,48,49].

## 5. Limitations

This study has several limitations, and its results should be interpreted with caution. The sample size was relatively small and purposive, limiting the generalizability of the findings to this specific demographic profile. Future studies should aim to recruit larger and more diverse samples. The retrospective and cross-sectional design of the study prevents establishing causal relationships between predictor variables and outcomes. Longitudinal studies are necessary to explore these causal links more thoroughly.

Additionally, the use of self-reported scales in this study may introduce bias. The study adopted a socio-health focus, relying on evaluations conducted by the Spanish Public Administration as a proxy for the level of disability in PwMS. While this approach aligns with local assessment practices, it does not utilize internationally recognized scales such as the EDSS or PDSS, making direct comparisons with other studies more difficult.

Moreover, we did not collect clinical data on PwMS, such as the number of relapses or disease activity, which are factors that can significantly influence caregiver burden. Future research investigating gender differences in the psychosocial functioning of MS caregivers should incorporate and analyze a broader range of clinical variables related to patients.

## 6. Conclusions

This study underscores the complexity of the role of male caregivers of PwMS. Along with the physical and emotional burdens commonly associated with informal caregiving, these men navigate challenges related to traditional gender stereotypes. While some experience gender dissonance, the study found that levels of self-stigma among male caregivers were generally low. Most participants employed strategies to reconcile caregiving with their masculine identity, whether by integrating caregiving into traditional masculine roles, adopting a gender-neutral perspective, or redefining masculinity to encompass caregiving tasks.

These findings highlight the adaptability of male caregivers in managing the interplay between caregiving and societal expectations of masculinity. They also emphasize the importance of developing tailored support interventions that address the unique experiences of male caregivers, help them navigate gendered challenges, and promote their overall well-being in the caregiving process.

## Figures and Tables

**Table 1:** Characteristics of caregivers and PwMS.

	Caregiver		PwMS	
	*n*/*M*	%/*SD*	*n/M*	%/*SD*
Gender				
Male	44	100	6	13.6
Female			38	86.4
Age	58.4	11.2	51.6	12.1
Relationship				
Spouse	32	72.7	32	72.7
Brother/sister	2	2.6	2	2.6
Father	6	13.6		
Mother			1	2.3
Child	1	2.3	6	13.6
Partner	3	6.8	3	6.8
Place of residence				
Almería	1	2.3	1	2.3
Cádiz	5	11.4	5	11.4
Córdoba	8	18.2	8	18.2
Granada	10	22.7	10	22.7
Huelva	4	9.1	4	9.1
Jaén	5	11.4	5	11.4
Málaga	4	9.1	4	9.1
Sevilla	7	15.9	7	15.9
Environment				
Rural	21	47.7	21	47.7
Urban	23	52.3	23	52.3
Marital status				
Single	3	6.8	6	13.6
Married, domestic partner	38	86.4	33	75.0
Separated/divorced	2	4.5	4	9.1
Widower	1	2.3	1	2.3
Housing				
Alone	3	6.8	2	4.6
Family of origin	1	2.3	0	0
Family formed	40	90.9	42	95.5
Employment				
Employed	20	45.5		
Unemployed	5	11.4		
Retired	19	43.2		
Education				
Primary	6	13.6		
Secondary	14	31.8		
Vocational Training 1	4	9.1		
Vocational Training 2	3	6.8		
Baccalaureate	5	11.4		
University	12	27.3		
His care is of the same quality as that of a woman	42	95.5		
MS pattern				
PP			2	4.5
PR			0	0
RR			25	56.8
SP			17	38.6
Disability			42	95.5
Level of disability				
None			2	4.5
33–65%			21	47.7
>65%			21	47.7
Reduced mobility			34	72.3
Dependency			20	45.5
Level of dependency				
None			24	54.5
Moderate			2	4.5
Severe			11	25
Great dependency			7	15.9

PP: primary progressive; PR: progressive relapsing; RR: relapsing–remitting; SP: secondary progressive.

**Table 2:** Caregiving Tasks in Multiple Sclerosis (CTiMS).

Activity/Care	*M*	*SD*
ADL CARE	1.2	1.2
1. Toileting	1.4	1.5
2. Feeding	0.7	1.1
3. Continence/diapers	0.9	1.5
4. Bathing	1.5	1.5
5. Dressing	1.5	1.5
6. Getting in/out of chairs	1.5	1.5
7. Giving medicine	1.2	1.5
PSYCHO-EMOTIONAL CARE	1.8	0.9
8. Managing the changes in patient’s personality	1.6	1.4
9. Managing the changes in patient’s mood swings/moodiness	1.9	1.2
10. Managing patient’s emotional difficulties	2.0	1.2
11. Helping patient with his/her memory difficulties	1.2	1.1
12. Managing patient’s fatigue	2.2	1.0
INSTRUMENTAL CARE	2.8	1.1
13. Grocery shopping	3.0	1.2
14. Housework	2.8	1.3
15. Preparing meals	2.6	1.4
16. Transportation	2.9	1.4
SOCIAL–PRACTICAL CARE	2.1	1.0
17. Managing finances/paying bills	2.2	1.6
18. Providing companionship to patient	2.5	1.3
19. Providing emotional support to patient	2.7	1.0
20. Making decisions for patient	2.2	1.3
21. Assiting patient with physical exercises	2.3	1.3
22. Working out what patient can and cannot do	1.9	1.1
23. Keeping patient occupied	1.3	1.2
24. Arranging supervision/outside services	2.1	1.6

0: never; 1: rarely; 2: sometimes; 3: often; 4: always.

**Table 3:** Caregiving burden (ZBI).

Burden	*n*	%
No-to-mild	0	0
Mild-to-moderate	14	31.8
Moderate-to-severe	25	56.8
Severe	5	11.4

No-to-mild burden: 0–20; mild-to-moderate burden: 21–40; moderate-to-severe burden: 41–60; severe burden: 61–88.

**Table 4:** Quality of life (WHOQOL).

Domain	*M*	*SD*	Min.	Max.
Quality of life (WHOQL1)	54.0	21.5	25	100
General health (WHOQL2)	52.8	22.4	25	100
Physical health	61.5	13.5	38	88
Psychological health	65.0	13.1	31	88
Social relationships	53.1	20.4	19	100
Environment	60.2	13.6	31	94

**Table 5:** Comparison of quality of life (WHOQOL) with caregiver burden (ZBI).

Domain	F (df)	*p*	η²
Quality of life (WHOQL1)	6.75 (2, 41)	0.0029	0.25
General health (WHOQL2)	0.75 (2, 41)	0.4780	0.04
Physical health	4.55 (2, 41)	0.0165	0.18
Psychological health	11.23 (2, 41)	0.0001	0.35
Social relationships	1.17 (2, 41)	0.3200	0.05
Environment	5.08 (2, 41)	0.0107	0.20

**Table 6:** Regression results using caregiver burden (ZBI) as the criterion.

Predictor	*b*	*b* [95% CI]	*sr^2^*	*sr^2^* [95% CI]	Fit
(Intercept)	55.94 **	[39.59; 72.30]			
RR	−23.89 **	[−40.57; −7.31]	0.17	[−0.03; 0.37]	
SP	−19.37 *	[−36.83; −1.90]	0.10	[−0.06; 0.26]	
33–65% disability	10.11	[−6.47; 26.69]	0.03	[−0.06; 0.12]	
>65% disability	12.88	[−4.35; 30.11]	0.05	[−0.06; 0.16]	
					*R^2^* = 0.220 *
					95% CI [0.00; 0.37]

* *p* < 0.05; ** *p* < 0.01.

**Table 7:** Regression results using quality of life (WHOQOL1) as the criterion.

Predictor	*b*	*b* [95% CI]	*sr^2^*	*sr^2^* [95% CI]	Fit
(Intercept)	44.39 **	[15.44; 73.34]			
RR	36.22 *	[6.87; 65.56]	0.10	[−0.04; 0.23]	
SP	37.78 *	[6.88; 68.69]	0.09	[−0.04; 0.23]	
33–65% disability	−13.78	[−43.13; 15.56]	0.01	[−0.04; 0.07]	
>65% disability	−39.84 *	[−70.33; −9.34]	0.11	[−0.04; 0.25]	
					*R^2^* = 0.404 **
					95% CI [0.12; 0.54]

* *p* < 0.05 **; *p* < 0.01.

**Table 8:** Task femininity.

Activity/Care	*M*	*SD*
ADL CARE	3.2	0.3
1. Toileting	3.3	0.6
2. Feeding	3.0	0.5
3. Continence/diapers	3.5	0.7
4. Bathing	3.5	0.6
5. Dressing	3.3	0.6
6. Getting in/out of chairs	2.9	0.5
7. Giving medicine	3.0	0.5
PSYCHO-EMOTIONAL CARE	3.1	0.4
8. Managing the changes in patient’s personality	3.1	0.5
9. Managing the changes in patient’s mood swings/moodiness	3.1	0.6
10. Managing patient’s emotional difficulties	3.2	0.6
11. Helping patient with his/her memory difficulties	3.0	0.4
12. Managing patient’s fatigue	2.9	0.5
INSTRUMENTAL CARE	3.3	0.5
13. Grocery shopping	3.3	0.8
14. Housework	3.6	0.7
15. Preparing meals	3.6	0.8
16. Transportation	2.7	0.6
SOCIAL-PRACTICAL CARE	2.9	0.3
17. Managing finances/paying bills	2.7	0.8
18. Providing companionship to patient	3.0	0.8
19. Providing emotional support to patient	3.1	0.6
20. Making decisions for patient	2.9	0.6
21. Assiting patient with physical exercises	2.8	0.6
22. Working out what patient can and cannot do	2.9	0.5
23. Keeping patient occupied	3.1	0.6
24. Arranging supervision/outside services	3.0	0.9

1: completely masculine; 2: quite masculine; 3: both masculine and feminine; 4: quite feminine; 5: completely feminine.

**Table 9:** Self-stigma and its components (SSS-S).

	*n*	%
Cognitive	7	15.9
My identity as a caregiver is a burden to me	8	18.2
My identity as a caregiver incurs inconvenience in my daily life	10	22.7
The identity of being a caregiver taints my life	8	18.2
Affective	0	0
I feel unconfortable because I am a caregiver	0	0
I fear that others would know that I am a caregiver	0	0
I feel like I cannot do anything about my caregiver status	10	22.7
Behavioral	0	0
I estrange myself from others because I am a caregiver	1	2.3
I avoid interacting with others because I am a caregiver	1	2.3
I dare not to make new friends lest they find out that I am a caregiver	0	0

## Data Availability

The datasets used and/or analyzed during the current study are available from the corresponding author upon reasonable request.

## References

[1] Brownlee W.J., Hardy T.A., Fazekas F., Miller D.H. (2017). Diagnosis of multiple sclerosis: Progress and challenges. Lancet.

[2] Rajachandrakumar R., Finlayson M. (2022). Multiple sclerosis caregiving: A systematic scoping review to map current state of knowledge. Health Soc. Care Community.

[3] Finlayson M., Cho C. (2008). A descriptive profile of caregivers of older adults with MS and the assistance they provide. Disabil. Rehabil..

[4] Corry M., While A. (2009). The needs of carers of people with multiple sclerosis: A literature review. Scand. J. Caring Sci..

[5] Topcu G., Buchanan H., Aubeeluck A., Garip G. (2016). Caregiving in multiple sclerosis and quality of life: A meta-synthesis of qualitative research. Psychol. Health.

[6] Opara J., Brola W. (2018). Quality of life and burden in caregivers of multiple sclerosis patients. Physiother. Health Act..

[7] Aymerich M., Guillamón I., Jovell A.J. (2009). Health-related quality of life assessment in people with multiple sclerosis and their family caregivers. A multicenter study in Catalonia (Southern Europe). Patient Prefer. Adherence.

[8] Labiano-Fontcuberta A., Mitchell A.J., Moreno-García S., Benito-León J. (2015). Anxiety and depressive symptoms in caregivers of multiple sclerosis patients: The role of information processing speed impairment. J. Neurol. Sci..

[9] Rivera-Navarro J., Morales-González J.M., Benito-León J., GEDMA (2003). Informal caregiving in multiple sclerosis patients: Data from the Madrid demyelinating disease group study. Disabil. Rehabil..

[10] García Calvente M., del Río Lozano M., Marcos Marcos J. (2011). Desigualdades de género en el deterioro de la salud como consecuencia del cuidado informal en España. Gac. Sanit..

[11] Bravo-Gonzalez F., Alvarez-Roldan A. (2019). Esclerosis múltiple, pérdida de funcionalidad y género. Gac. Sanit..

[12] Pakenham K.I. (2007). The nature of caregiving in multiple sclerosis: Development of the caregiving tasks in multiple sclerosis scale. Mult. Scler..

[13] Martín-Carrasco M., Otermin P., Pérez-Camo V., Pujol J., Agüera L., Martín M., Gobartt A., Pons S., Balañá M. (2010). EDUCA study: Psychometric properties of the Spanish version of the Zarit Caregiver Burden Scale. Aging Ment. Health.

[14] Zarit S.H., Reever K., Bahc-Peterson J. (1980). Relatives of the impaired elderly: Correlates of feelings of burden. Gerontologist.

[15] Martín-Carrasco M., Domínguez-Panchón A.I., Muñoz-Hermoso P., González-Fraile E., Ballesteros-Rodríguez J. (2013). Instrumentos para medir la sobrecarga en el cuidador informal del paciente con demencia. Rev. Esp. Geriatr. Gerontol..

[16] Skevington S.M., Lotfy M., O’Connell K.A. (2004). The World Health Organization’s WHOQOL-BREF quality of life assessment: Psychometric properties and results of the international field trial A Report from the WHOQOL Group. Qual. Life Res..

[17] The WHOQOL Group (1998). WHO Quality of Life Scale (WHOQOL). Psychol. Med..

[18] Lucas-Carrasco R. (2012). The WHO quality of life (WHOQOL) questionnaire: Spanish development and validation studies. Qual. Life Res..

[19] Lucas-Carrasco R., Laidlaw K., Power M.J. (2011). Suitability of the WHOQOL-BREF and WHOQOL-OLD for Spanish older adults. Aging Ment. Health.

[20] Burgwal A. (2020). Measuring Gender Identity: A Comparison Between the Likert Scale and the Fuzzy Scale [Internet].

[21] Mak W.W.S., Cheung R.Y.M. (2010). Self-stigma among concealable minorities in Hong Kong: Conceptualization and unified measurement. Am. J. Orthopsychiatry.

[22] Maguire R., Maguire P. (2020). Caregiver burden in multiple sclerosis: Recent trends and future directions. Curr. Neurol. Neurosci. Rep..

[23] García-Calvente M., Marcos-Marcos J., del Río-Lozano M., Hidalgo-Ruzzante N., Maroto-Navarro G. (2012). Embedded gender and social changes underpinning inequalities in health: An ethnographic insight into a local Spanish context. Soc. Sci. Med..

[24] del Río-Lozano M., García-Calvente M.d.M., Marcos-Marcos J., Entrena-Durán F., Maroto-Navarro G. (2013). Gender identity in informal care: Impact on health in Spanish caregivers. Qual. Health Res..

[25] Parry M. (2019). Caregiver burden and cardiovascular disease: Can we afford to keep the health of caregivers in Canada invisible?. Can. J. Cardiol..

[26] Ferrant G., Pesando L.M., Nowacka K. (2014). Unpaid Care Work: The Missing Link in the Analysis of Gender Gaps in Labour Outcomes.

[27] Glauber R. (2017). Gender differences in spousal care across the later life course. Res. Aging.

[28] del Río Lozano M., García-Calvente M.M., Calle-Romero J., Machón-Sobrado M., Larrañaga-Padilla I. (2017). Health-related quality of life in Spanish informal caregivers: Gender differences and support received. Qual. Life Res..

[29] Rodríguez-Madrid M.N., Del Río-Lozano M., Fernández-Peña R., García-Calvente M.D.M. (2021). Changes in caregiver personal support networks: Gender differences and effects on health (cuidar-se study). Int. J. Environ. Res. Public Health.

[30] Comas-d’Argemir D., Soronellas M. (2019). Men as carers in long-term caring: Doing gender and doing kinship. J. Fam. Issues.

[31] Comas-d’Argemir D. (2016). The involvement of men in care. Men’s experiences as family. Irish J. Anthropol..

[32] McKenzie T., Quig M.E., Tyry T., Marrie R.A., Cutter G., Shearin E., Johnson K., Simsarian J. (2015). Care partners and multiple sclerosis: Differential effect on men and women. Int. J. MS Care.

[33] Lopez–Anuarbe M., Kohli P. (2019). Understanding male caregivers’ emotional, financial, and physical burden in the United States. Healthcare.

[34] Ponzio M., Tacchino A., Verri A., Battaglia M.A., Brichetto G., Podda J. (2024). Profile and burden of the family caregiver: The caring experience in multiple sclerosis. An observational study. BMC Psychol..

[35] Benito-León J., Rivera-Navarro J., Guerrero A.L., Heras V.d.L., Balseiro J., Rodríguez E., Belló M., Martínez-Martín P. (2011). The CAREQOL-MS was a useful instrument to measure caregiver quality of life in multiple sclerosis. J. Clin. Epidemiol..

[36] Buhse M., Ratta CDella Galiczewski J., Eckardt P. (2015). Caregivers of older persons with multiple sclerosis: Determinants of health-related quality of life. J. Neurosci. Nurs..

[37] Bayen E., Papeix C., Pradat-Diehl P., Lubetzki C., Joël M.E. (2015). Patterns of objective and subjective burden of informal caregivers in multiple sclerosis. Behav. Neurol..

[38] García-Calvente M.d.M., Hidalgo-Ruzzante N., del Río-Lozano M., Marcos-Marcos J., Martínez-Morante E., Maroto-Navarro G., Mateo-Rodríguez I., Gil-García E. (2012). Exhausted women, tough men: A qualitative study on gender differences in health, vulnerability and coping with illness in Spain. Sociol. Health Illn..

[39] Bueno M.V. (2024). A duty to care: Male perspectives on the caregiver role for persons with Alzheimer’s or dementia. J. Fam. Nurs..

[40] Bueno M.V., Chase J.A.D. (2023). Gender differences in adverse psychosocial outcomes among family caregivers: A systematic review. West. J. Nurs. Res..

[41] Peña-Longobardo L.M., Río-Lozano MDel Oliva-Moreno J., Larrañaga-Padilla I., García-Calvente M.D.M. (2021). Health, work and social problems in Spanish informal caregivers: Does gender matter? (the Cuidar-Se study). Int. J. Environ. Res. Public Health.

[42] Swinkels J., Tilburg T., Van Verbakel E., Van Groenou M.B. (2019). Explaining the gender gap in the caregiving burden of partner caregivers. J. Gerontol B Psychol. Sci. Soc. Sci..

[43] Rollero C. (2016). The experience of men caring for a partner with multiple sclerosis. J. Nurs. Sch..

[44] Hellström I., Håkanson C., Eriksson H., Sandberg J. (2017). Development of older men’s caregiving roles for wives with dementia. Scand. J. Caring Sci..

[45] Mott J., Schmidt B., MacWilliams B. (2019). Male caregivers. Shifting roles among family caregivers. Clin. J. Oncol. Nurs..

[46] Leung L.C., Chan K.W., Tam K.Y. (2019). Reconstruction of masculine identities through caring practices: The experiences of male caregivers in Hong Kong. J. Fam. Issues.

[47] Medved C.E. (2016). Stay-at-home fathering as a feminist opportunity: Perpetuating, resisting, and transforming gender relations of caring and earning. J. Fam. Commun..

[48] Björk S. (2015). Doing, re-doing or undoing masculinity? Swedish men in the filial care of aging parents. NORA—Nord. J. Fem. Gend. Res..

[49] Hunter S.C., Riggs D.W., Augoustinos M. (2017). Hegemonic masculinity versus a caring masculinity: Implications for understanding primary caregiving fathers. Soc. Pers. Psychol. Compass.

